# Clinicoradiological changes of brain NK/T cell lymphoma manifesting pure akinesia: a case report

**DOI:** 10.1186/1471-2377-11-137

**Published:** 2011-11-02

**Authors:** Susumu Ishihara, Osamu Kano, Ken Ikeda, Reiko Shimokawa, Kiyokazu Kawabe, Yasuo Iwasaki

**Affiliations:** 1 Department of Hematology and Oncology, Toho University Omori Medical Center, Tokyo, Japan; 2 Department of Neurology, Toho University Omori Medical Center, Tokyo, Japan; 3 Division of Pathology, Saiseikai Yokohamashi Tobu Hospital, Yokohama, Japan

## Abstract

**Background:**

Pure akinesia (PA) is a distinct form of parkinsonism characterized by freezing phenomena. Little is known about brain tumor-associated PA. We highlight the clinicoradiological changes in a patient with PA and central nervous system (CNS) metastases of natural killer/T-cell lymphoma (NKTL).

**Case presentation:**

A 68-year-old man with stage IVB extranodal NKTL developed a gait disturbance. Neurological examination of his gait revealed freezing, start hesitation, short step, forward flexion posture, festination and postural instability. Mild facial hypomimia and micrographia were observed. There was no rigidity or tremor in any of the four extremities. Brain magnetic resonance imaging (MRI) displayed T2-hyperintense lesions in the dorsal brainstem, cerebellum and periventricular white matter. Diffusion-weighted imaging (DWI) and the apparent diffusion coefficient (ADC) revealed hyperintensity in these regions. Cerebrospinal fluid cytology revealed CD56-positive cells on immunohistochemical staining. The patient's neurological deficits did not respond to L-dopa treatment and intrathecal administration of methotrexate (MTX). Two weeks later, he displayed confusion and generalized convulsions. T2-hyperintense lesions spread to the basal ganglia and the infratentorial regions. Gadolinium enhancement was observed in the cerebellum and frontal subcortex. DWI and the ADC revealed diffusion-restricted lesions in the middle cerebellar peduncles, left internal capsules and cerebral white matter. MTX pulse therapy and intrathecal administration of cytosine arabinoside and MTX were performed. Two months later, his ambulatory state was normalized. Brain MRI also revealed marked alleviation of the infratentorial and supratentorial lesions.

**Conclusions:**

The clinicoradiological profile of our patient suggested that dorsal ponto-mesencephalic lesions could contribute to the pathogenesis of PA. Physicians should pay more attention to striking CNS seeding of metastatic NKTL. MTX pulse therapy had an excellent effect in improving serious symptoms and brain lesions in our patient.

## Background

Pure akinesia (PA) is a distinct form of parkinsonism that was first reported in 1972 as freezing phenomena only, characterized by frozen gait, micrographia and festinating speech [1]. Limb rigidity and tremor were not observed. L-dopa treatment had no effects in PA patients [2,3]. Many studies of neoplastic parkinsonism have been reported [4-14]. However, little is known about brain tumor-associated PA [4,5]. We highlight the clinicoradiological changes in a unique patient with PA and central nervous system (CNS) metastasis of natural killer/T cell lymphoma (NKTL).

## Case presentation

**Figure 1:**
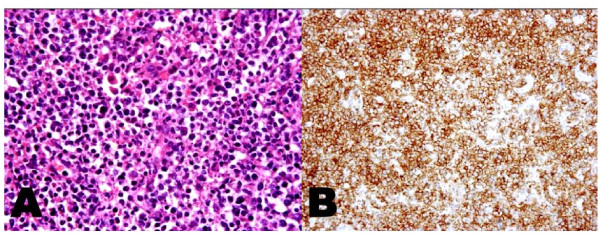
**Biopsy specimen of the adrenal gland**. **A**. Hematoxylin and eosin staining. Lymphoid cells are characterized by an intermediate size and nuclei with irregular contours and inconspicuous nucleoli. Original magnification ×200. **B**. Immunohistochemical CD56 staining. Most cell membranes are CD56 positive. Original magnification ×200.

A 68-year-old man developed anorexia and exhibited body weight loss for 2 months. Abdominal computed tomography showed massive lesions in the adrenal glands. Laparoscopic biopsy of the adrenal gland demonstrated a pathological diagnosis of extranodal NKTL (Figure 1). Clinical stage IVB was confirmed. Two courses of DeVIC (carboplatin, etoposide, ifosfamide and dexamethasone) combination chemotherapy led to marked amelioration of the pericardial, pulmonary and pancreatic lesions. Two weeks later, the patient developed a gait disturbance and came to our neurology department. Physical examination showed blood pressure of 120/78 mm Hg and body temperature of 36.2°C. His level of consciousness and cognitive function were normal. Neurological examination revealed a frozen gait with start hesitation, short step, forward flexion posture, festination and postural instability. Kinesia paradoxale was observed when obstacles were placed in his path. He had mild facial hypomimia, reduced blinking, slight slurred speech and micrographia. There was no limb rigidity or tremor in the four extremities. The remaining examination was normal. These neurological observations supported the clinical diagnosis of PA.

**Figure 2:**
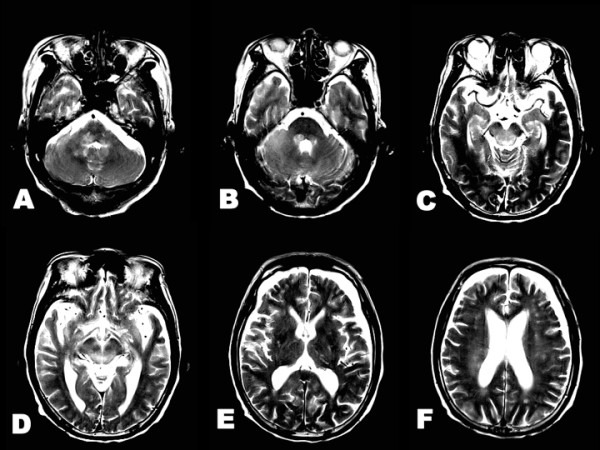
**Brain T2-weighted imaging at neurological onset**. **A-F**. Hyperintense lesions are observed in the dorsal pons, midbrain, cerebellum and periventricular white matter. **E**. There were no remarkable changes in the basal ganglia.

Routine laboratory studies were normal. Serum soluble interleukin-2 receptor levels increased to 1850 U/mL (normal range: 220-530). There were no paraneoplastic antibodies, including antineuronal nuclear antibody types 1, 2 and 3. Serum anti-human immunodeficiency virus antibodies were not detected. Cerebrospinal fluid (CSF) analyses showed a mononuclear cell count of 28/mm^3 ^and total protein levels of 138 mg/dL. CSF cytology revealed CD56-positive cells on immunohistochemical staining. Infectious pathogen tests for herpes simplex, tuberculosis, bacteria and fungus were all negative. Brain magnetic resonance imaging (MRI) revealed T2-hyperintense lesions in the dorsal brainstem, cerebellum and periventricular white matter. Abnormal T2-intensities were not found in the basal ganglia (Figure 2). Diffusion-weighted imaging (DWI) and the apparent diffusion coefficient (ADC) revealed a mild to moderate degree of hyperintensity in the brainstem and cerebellum.

**Figure 3:**
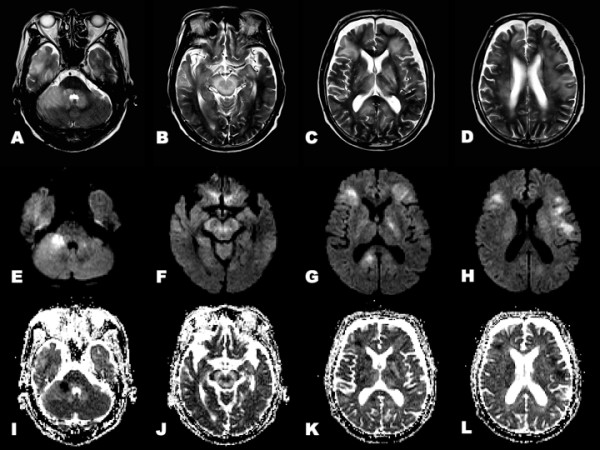
**Brain MRI after neurological worsening**. **A-D**. T2-weighted image show widespread T2-hyperintense lesions in the brainstem, cerebellum, basal ganglia and cerebral white matter. **E-H**. DWI shows hyperintense lesions in the middle cerebellar peduncles, midbrain, left internal capsules and cerebral white matter. **I-L**. ADC shows hypointense lesions in the middle cerebellar peduncles, midbrain, left internal capsules and cerebral white matter.

**Figure 4:**
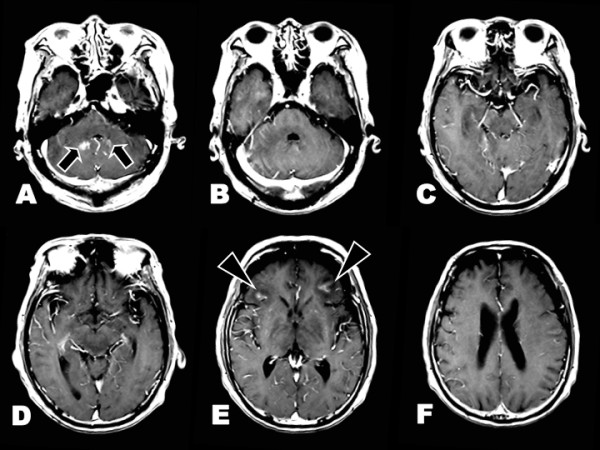
**Gadolinium-enhanced T1-weighted imaging**. **A, E**. Gadolinium enhancement are found in the cerebellum (arrows) and bilateral frontal subcortex (arrowheads). **B-D, F**. There is no enhancement in the brainstem and basal ganglia.

**Figure 5:**
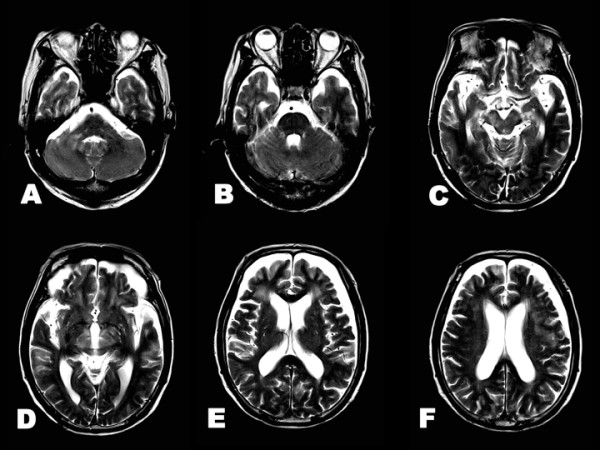
**Brain T2-weighted imaging after neurological recovery**. **A-F**. T2-hyperintense lesions are markedly attenuated. Hyperintense lesions remain in the right middle cerebellar peduncle, midbrain and cerebral white matter.

Neither intrathecal administration of cytosine arabinoside (Ara-C), methotrexate (MTX) and prednisolone (PSL), nor L-dopa treatment (levodopa 300 mg/day and carbidopa 30 mg/day), had any effect on the patient's PA. Two weeks later, the patient experienced confusion and a generalized tonic seizure. Brain MRI revealed widespread T2-hyperintense lesions in the infratentorial region and basal ganglia (Figure 3A-D). DWI and ADC disclosed restricted water diffusivity in the middle cerebellar peduncles, brainstem, left internal capsules and cerebral white matter (Figure 3E-L). Gadolinium enhancement was found in the cerebellum and bilateral frontal subcortex. There was no abnormal enhancement in the brainstem and basal ganglia (Figure 4). The patient's level of consciousness deteriorated, and he became bedridden. After intravenous MTX pulse therapy (4500 mg/day) and the second intrathecal administration of Ara-C, MTX and PSL, he recovered to normal consciousness and became ambulatory with support. He had no hypertensive episodes during the entire course. Two months later, PA was ameliorated completely without L-dopa treatment. CSF cytology showed no CD56-positive cells. T2-hyperintense lesions were markedly attenuated in the brainstem, basal ganglia and cerebral white matter (Figure 5). DWI and ADC showed remarkable alleviation of diffusion-restricted lesions in the middle cerebellar peduncles, brainstem and cerebral white matter.

## Discussion

We reported a patient with CNS metastases of NKTL who had a transient course of PA and striking changes in the brain lesions.

The frequency of CNS metastases is 5-9% in patients with systemic non-Hodgkin lymphoma. Most patients have leptomeningeal or spinal epidural metastases. The rate of CNS parenchymal metastases is only 1% [15]. NKTL is a rare type of lymphoma that usually presents as destructive lesions within the nasal cavity. Nasal NKTL frequently involves extranodal sites such as the lung, skin and CNS [16]. CNS invasion or metastasis is common in patients with nasal NKTL [17]. Most NKTL patients have aggressive courses and unfavorable clinical outcomes. During PA onset, our patient showed lesions in the brainstem and periventricular white matter on MRI. After he exhibited confusion and generalized convulsion, T2-hyperintense lesions were observed in the middle cerebellar peduncles and basal ganglia. DWI and the ADC revealed gadolinium-enhanced and -nonenhanced diffusion-restricted lesions in the middle cerebellar peduncles, brainstem and cerebral white matter. These neuroradiological features suggested the rapid relapse of brain NKTL. Extensive MRI lesions in the cerebral white matter have previously been described in a patient with nasal NKTL [18]. Recent neuroradiological studies have pointed out the inverse relationship between ADC and the cell density of B cell lymphoma [19,20]. The clinicoradiological worsening of our patient might have resulted from the fulminant progression of CNS metastases of NKTL and cerebral edema.

As mentioned previously, many studies of neoplastic parkinsonism have been reported [4-14]. The following pathogenic mechanisms underlying parkinsonism induced by brain tumors have been proposed: 1) damage of striatal postsynaptic cells in the basal ganglia or substantia nigra secondary to mechanical pressure or intrinsic involvement; 2) impairment of the pathway between the striatum and the supplementary motor area; and 3) compression and/or distortion of the nigrostriatal pathway. The topographic distribution of the brain tumors is divided into the supratentorial region and brainstem. Compared with supratentorial tumors, the frequency of brainstem tumor-related parkinsonism is rare. Previous reports of neoplastic parkinsonism associated with the midbrain lesion are summarized in Table 1. The patterns of parkinsonism onset and the responses to L-dopa were highly variable. In these reports, the pathological diagnosis was astrocytoma in three patients and B cell lymphoma in three patients. The present patient was diagnosed as CNS metastases of NKTL.

**Table 1:** Previous literature of brainstem tumor-related parkinsonism

Authors [*] (years)	Age/gender	Histological diagnosis	Tumor location	Onset form	L-dopa treatment
Gherardit et al [9] (1985)	59 years/male	B cell lymphoma	Midbrain, thalamus	Acute	Excellent response
Cicarelli et al [10] (1999)	39 years/female	Astrocytoma	Pons, midbrain	Unknown	No response
Yoshimura et al [11] (2002)	63 years/female	Astrocytoma	Midbrain	Chronic	Excellent response
Lin et al [12] (2010)	81 years/male^†^	B cell lymphoma	Midbrain	Acute	No response
Hatano et al [13] (2011)	70 year/male	B cell lymphoma	Midbrain, hypothalamus, pineal body, thalamus, pallidum	Acute	No administration
Wächter et al [14] (2011)	74 years/male	Astrocytoma	Pons, midbrain, thalamus	Chronic	Moderate response
Present patient	68 years/male	NKTL	Pons, midbrain, cerebellum cerebral white matter	Acute	No response

*Reference number.

^† ^Complication of obstructive hydrocephalus.

With respect to the pathogenesis of PA, previous pathological studies have shown that some PA patients had similar lesions to those of progressive supranuclear palsy (PSP) patients [21-23]. Neuroradiological studies have also reported matching results between PA and PSP patients [24,25]. Only two patients with neoplastic PA have been reported [4,5]. Suzuki et al. [4] described a 44-year-old man with primary CNS reticulum cell sarcoma. Pramstaller et al. [5] reported the case of a 75-year-old man with primary CNS B cell lymphoma. This patient did not respond to L-dopa treatment, and autopsy demonstrated the involvement of the bilateral globus pallidus [5]. The precise pathophysiological mechanism of PA remains unclear. In general, PA might contribute to both presynaptic and postsynaptic damage in nigrostriatal dopaminergic neurons [24,25]. The midbrain tegmentum is the most common lesion site between PA and PSP patients [21-23]. A dorsorostral midbrain lesion was reported to cause PSP-like symptoms in a patient who had normal nigrostriatal dopaminergic function after resection of a pineal gland tumor [26]. Therefore, the neuroradiological changes of our patient supported the concept that damage of the ponto-mesencephalic tegmentum could contribute to the pathogenesis of PA.

## Conclusion

We highlighted the clinicoradiological course in a patient with transient PA and CNS metastases of NKTL. The neuroradiological changes of the present patient suggest that physicians should pay more attention to fulminant progression of CNS metastases in NKTL patients. MTX pulse therapy dramatically improved the serious neurological deficits and brain lesions in our patient.

## Consent

Informed consent was obtained from the patient and his spouse for publication of this case report and any accompanying images.

## Competing interests

The authors declare that they have no competing interests.

## Authors' contributions

KI participated in the clinicoradiological studies, its design and drafted the manuscript. SI and KK carried out the clinical evaluation of the patient and wrote the patient case. OK carried out the neuroradiological studies. RS carried out the pathological analysis. YI participated in coordination and helped to draft the manuscript. All authors read and approved the final manuscript.

## Pre-publication history

The pre-publication history for this paper can be accessed here:

http://www.biomedcentral.com/1471-2377/11/137/prepub
